# A genome-wide collection of barcoded single-gene deletion mutants in *Salmonella enterica* serovar Typhimurium

**DOI:** 10.1371/journal.pone.0298419

**Published:** 2024-03-07

**Authors:** Steffen Porwollik, Weiping Chu, Prerak T. Desai, Michael McClelland

**Affiliations:** 1 Dept. of Microbiology and Molecular Genetics, University of California, Irvine, Irvina, CA, United States of America; 2 GSK Computational Biology, Upper Providence, PA, United States of America; Gandhi Insititute of Technology and Management, INDIA

## Abstract

Genetic screening of pools of mutants can reveal genetic determinants involved in complex biological interactions, processes, and systems. We previously constructed two single-gene deletion resources for *Salmonella enterica* serovar Typhimurium 14028s in which kanamycin (Kan^R^) and chloramphenicol (Cam^R^) cassettes were used to replace non-essential genes. We have now used lambda-red recombination to convert the antibiotic cassettes in these resources into a tetracycline-resistant (Tet^R^) version where each mutant contains a different 21-base barcode flanked by Illumina Read1 and Read2 primer sequences. A motility assay of a pool of the entire library, followed by a single-tube processing of the bacterial pellet, PCR, and sequencing, was used to verify the performance of the barcoded Tet^R^ collection. The new resource is useful for experiments with defined subsets of barcoded mutant strains where biological bottlenecks preclude high numbers of founder bacteria, such as in animal infections. The Tet^R^ version of the library will also facilitate the construction of triple mutants by transduction. The resource of 6197 mutants covering 3490 genes is deposited at Biological and Emerging Infections Resources (beiresources.org).

## Introduction

*Salmonella* is a common foodborne enterobacterium that has been intensely studied in medical research. The species can be easily cultured in standard laboratory conditions, adding to its popularity as a basic research organism. Because of its versatility as a human pathogen, it can be used in different models to study gastrointestinal infection as well as invasive forms of infections [1–5]. Decades of research have made *Salmonella* one of the most understood bacterial pathogens. Nevertheless, functions have not yet been characterized for many of its genes, and novel roles are regularly identified for genes that were previously characterized. Numerous genetic determinants of the bacterium’s survival in countless environments are still unknown.

Genetic screening of pools of mutants is one of the oldest methods to reveal genetic determinants involved in complex biological interactions, processes and systems. The caveats, such as partially compensatory mutations, genetic redundancies, and communal dependencies, are well known and methods to manage and even exploit them have been developed over decades [6]. The development of mutagenesis approaches based on the random insertion of transposons, with accompanying PCR and sequencing of the flanking regions, allowed for genome-wide genetic screening of pools of mutant libraries [7]. The incorporation of barcodes into the transposons, pioneered as signature-tagged mutagenesis [8], can streamline downstream analysis and was used by many research groups to generate mutant pools in dozens of bacterial pathogens [9]. In particular, barcoding can remove the requirement for multistep preparation of flanking bacterial DNA regions for sequencing and allows protocols that use a simple PCR-based sample preparation procedure followed by Illumina sequencing of the barcode, such as the one we have developed [10]. To date, we have made barcoded random transposon-based libraries in eight *Salmonella* serovars and in *E*. *coli* Nissle 1917 (**S1 Table**) [10, 11].

Pools of random transposon mutants suffer from an important limitation–in order to obtain representation of most or all non-essential genes, such libraries have to be generated and utilized at high complexities, precluding successful experiments in biological systems where the number of surviving founder bacterial clones that colonize certain niches is restricted. These biological bottlenecks are important in almost all infection studies. One strategy to address this issue is to create an ordered collection of mutants where the genomic locations of the transposon insertions are mapped and known for each clone. Ordered transposon-based libraries exist for a number of important bacterial pathogens including *Staphylococcus aureus* [12, 13], *Klebsiella pneumoniae* [14], uropathogenic *E*. *coli* [15], *Enterococcus faecalis* [16], *Vibrio cholerae* [17], *Pseudomonas aeruginosa* [18, 19], *Mycobacterium bovis* [20, 21], and *Borrelia burgdorferi* [22]. We have created such ordered resources for *Salmonella* serovars Enteritidis, and Choleraesuis, and for *E*. *coli* Nissle 1917 (**S1 Table**). Our ordered resources include unique characterized barcodes flanked by Illumina Read sequences, allowing easy single-tube processing and PCR of the bacterial pellet after selection experiments. In addition to combining maximal genetic coverage with minimal complexity, specific subsets of ordered mutants (few enough to successfully traverse a bottleneck in the environment of interest) can be readily assembled.

A third type of genome-wide mutant resource are single-gene deletion (SGD) mutant collections for all non-essential genes present on a bacterial genome. These have many advantages, including the lack of potential residual gene function inherent in insertion mutants and the ability to assemble clean deletion mutant sets for further study, to confirm mutations with an interesting phenotype in genetic screens. The first genome-wide SGD collection in bacteria was constructed for *E*. *coli* [23, 24], and other single-gene deletion libraries exist in model bacteria such as *Bacillus subtilis* [25] and *Helicobacter pylori* [26]. Our previously reported SGD collections of *Salmonella enterica* sv Typhimurium 14028s (STM14028s), a pathogenic strain of an important non-typhoidal *Salmonella* serovar, cover 71% of all genes and contain 3508 CDS-targeting kanamycin-resistant (Kan^R^) mutants and 3367 chloramphenicol-resistant (Cam^R^) clones [27, 28]. The existence of two separate clone lineages and resistance cassettes in this resource allows easy construction of double mutants by. The collections have been used in numerous published studies to identify genes of functional importance, such as in mice [29], chicks [30], epithelial cells [31], and T cells [32], and to investigate the mode of action of antimicrobials [33], the formation and revival of persisters [34], and the mode of action of antibiofilm compounds [35].

Here, we have converted the mutants of our STM14028s SGD collections to introduce a 21-base random barcode flanked by Illumina Read 1 and Read 2 sequences into each clone, facilitating easier downstream analysis by standard Illumina sequencing. To this end, we replaced the existing antibiotic cassettes with a barcoded tetracycline resistance (Tet^R^) cassette containing Illumina Read primer sequences. To build this resource of barcoded mutants, we performed seven rounds of recombination, mapping, and cherry-picking of unconverted mutants on each of the two collections (Kan^R^ and Cam^R^). The new barcoded Tet^R^ SGD mutant set covers over 90% of the genes represented by the previously existing, non-barcoded, collections of mutants.

## Materials and methods

### Primers

**Table 1** lists the primers used in this study.

**Table 1:** Primers used for the conversion and analysis of non-barcoded SGD Kan^R^ and Cam^R^ mutants to barcoded Tet^R^ clones.

Number	Primer name	When used?	Primer sequence
1	UL1	PCR1_P1_L	CACCAAACACCCCCCAAAACC
2	UR1	PCR1_P1_R	CACACAACCACACCACACCAC
3	Cassette_Right_Read1_N21_cRead2_UL1	PCR2_P1_L and PCR4_P1_L	GTGCAGGCTGGAGCTGCTTCGAAGTTCCTATACTTTCTAGAGAACACTCTTTCCCTACACGACGCTCTTCCGATCTNNNNNNNNNNNNNNNNNNNNNAGATCGGAAGAGCACACGTCTGAACTCCAGTCACCACCAAACACCCCCCAAAACC
4	Cassette_Left_UR1	PCR2_P1_R and PCR4_P1_R	CATATGAATATCCTCCTTAGGTCTCCCTATAGTGAGTCGTATTAATTTCGGAATTCCTATTCCGAAGTTCCTATTCTCTAGAAAGTATAGGAACTTCNNNNNNNNNNNNNNNNNNNNNCACACAACCACACCACACCAC
5	Nested_Primer_1	PCR3_P1_L	TTTCCCTACACGACGCTCTT
6	Nested_Primer_2	PCR3_P1_R	CGGAATTCCTATTCCGAAGTT
7	Klenow_N10	Klenow1_P1	GGCCATTAATACGACTCACTATAGGGAGACCGGCCTNNNNNNNNNN
8	P5_attachment	Klenow2_P1_L	AATGATACGGCGACCACCGAGATCTACACGGCCATTAATACGACTCACTATAGGGAGACC
9	Standard_indexed_Read2_engraftment	Klenow2_BC_P7_Index_Read2	CAAGCAGAAGACGGCATACGAGAT index (8mer) GTGACTGGAGTTCAGACGTGTGCTCTTCCGATCT
10	Standard_indexed_Read1_engraftment	BC_P5_Index_Read1	AATGATACGGCGACCACCGAGATCTACAC index (8mer) ACACTCTTTCCCTACACGACGCTCTTCCGATCT

### Generation of the PCR product for the conversion of non-barcoded *S*. *enterica* 14028s mutants

*Salmonella enterica* sv Typhimurium LT2 strain TT25401 contains a universal Tn10-derived tetracycline resistance cassette Tet^R^ (*https*:*//rothlab*.*ucdavis*.*edu/drugs/tetra*.*shtml*). Genomic DNA from this strain, a generous gift from John R. Roth, was used as a template in a PCR reaction with primers *UL1* and *UR1* which amplified the Tet^R^ cassette. Here and throughout, unless otherwise specified, PCR was performed for 35 cycles with 4μM of each primer in a volume of 25μl using 0.5 units of Q5 DNA polymerase in recommended buffer (NEB) with 10 sec of 98°C denaturation, 60°C annealing for 60 seconds, 72°C extension for 30 sec, with a final extension of 72°C for 5 minutes, followed by a hold at 8°C. A subsequent PCR on the amplified product PCRP1 utilized primers *Cassette_Right_Read1_N21_cRead2_UL1* and *Cassette_Left_UR1* that introduced sequences homologous to the ends of the resistance modules present in the non-barcoded Cam^R^ and Kan^R^ mutants as well as Illumina Read 1 and Read 2 segments flanking a random N_21_ barcode. The resulting PCR product PCRP2 was then electroporated into cells of an existing SGD Cam^R^ *aadA* mutant that was made electrocompetent by standard procedures (three washes of a logarithmically growing cell culture in an ice-cold solution of decreasing glycerol content) to yield barcoded tetracycline-resistant transformants.

The Tet^R^ cassette introduced into this mutant was amplified from its genomic DNA using two primers, *Nested_Primer_1* and *Nested_Primer_2*, that amplified almost all of the cassette. PCRP3 was then used as template in a final PCR reaction using primers *Cassette_Right_Read1_N21_cRead2_UL1* and *Cassette_Left_UR1*. The conditions were standard except that after five cycles the annealing and extension were set to 72°C. The resulting PCR product PCRP4 was then utilized on competent cells made from the pool of mutants of the non-barcoded Cam^R^ collection and, separately, the pool of mutants of the non-barcoded Kan^R^ collection.

The reason for the additional third PCR was to ensure that large amounts of DNA template were available for the final PCR that introduced the N21 barcodes. The primers used (Nested_Primer_1 and Nested_Primer_2) were small such that any primer dimer was easily removed. Furthermore, the subsequent step was nested, such that any residual dimer could not be amplified by the final primers. Not using this intermediate third PCR step resulted in a large amount of primer dimer from the genomic DNA.

### Conversion of non-barcoded SGD mutants to barcoded SGD mutants

Tetracycline-resistant mutants obtained after electroporation of PCRP4 into competent Cam^R^ or Kan^R^ SGD mutant pools were picked from selective LB agar plates supplemented with tetracycline at 15 μg/ml into 96-well plates, tested for remaining kanamycin or chloramphenicol resistance by replica-plating onto kanamycin and chloramphenicol plates, and mutants that were tetracycline resistant but kanamycin and chloramphenicol sensitive were pooled. Pools were then subjected to a Klenow amplification of the genomic region bordering the insertion site, and sequenced (see below), to determine which of the existing non-barcoded mutants had been successfully converted. Successfully converted Kan^R^ (SGDK) and Cam^R^ (SGDC) mutants were subsequently removed from the ordered collection of non-barcoded mutants (using a Beckman Biomek FX liquid handling robot), and the remaining (not yet converted) mutants were pooled again, made competent, and subjected to electroporation with PCRP4, as above. In seven iterations of this process, over 90% of the existing non-barcoded mutants were successfully converted.

### Determination of insertion sites of converted single-gene deletion mutants

Insertion sites of converted SGD mutants were determined via Klenow amplification and subsequent PCR, followed by Illumina sequencing, essentially as previously described [28]. Briefly, genomic DNA of mutant pools was prepared (GenElute Bacterial Genomic DNA kit, Sigma), denatured for 5 min at 95°C, randomly primed with 0.2μM *Klenow_N10*, a primer that contains a constant region followed by ten 3’ N bases, and extended with 50 U exo^-^ Klenow enzyme (New England Biolabs). After a gradual increase of temperature over 6 min to 37°C, the reaction proceeded for 30 min. Following enzyme deactivation at 75°C for 20 min and subsequent QIAquick purification (Qiagen), the 5′ (barcoded) product ends were amplified by PCR using 2.5 U Q5 enzyme (New England Biolabs) and 0.2μM of *P5_attachment* primer that contains the P5 engraftment sequence of Illumina with the 3’ end being identical to the constant region of *Klenow_N10*, and one of several standard Illumina primers for attaching the Read 2 P7 end with an 8-base index. Amplification proceeded using the following program: 94°C for 3 min, 15 cycles of 94°C for 15 sec, 58°C for 30 sec, and 72°C for 1 min, and a final extension at 72°C for 5 min. Products were QIAquick purified (Qiagen) and assembled at roughly equimolar amounts for sequencing.

The products were then paired-end sequenced using 150-base reads (Illumina). Read 2 sequences that mapped to the genome were identified using CLC Genomics Workbench (Qiagen). Subsequently, Read 2 sequences that contained the 21-base barcode, a constant region, and then 40 bases of the *Salmonella* genome were parsed into barcodes along with the associated adjacent *Salmonella* genomic sequence. For each barcode, the 5’ base positions, the frequency at that position, and the orientation in the 14028s genome were determined. Hits were filtered for quality, and for mapping to near an expected junction in the correct genome orientation of known mutants in the non-barcoded collections, thereby identifying the barcode(s) associated with each mutant.

### Pooling to identify plate/well locations of barcoded mutants and sequencing of barcodes

Pooling to identify plate/well locations of each of the picked mutant clones was done robotically, using a Biomek FX (Beckman). All mutants from each picked plate were pooled (66 *plate pools* in total) and all mutants from the same well from all plates of mutants converted from non-barcoded Kan^R^ or non-barcoded Cam^R^ were pooled, separately (95 *well pools* each for a total of 190 *well pools*, wells H12 had remained empty on all plates).

The identity of each barcode was determined by Illumina sequencing, essentially as described [10]. Briefly, the cells were washed three times in water, and digested with proteinase K (100 ug) for 2h at 55°C in lysis buffer (10 mM Tris [pH 8.0], 1 mM EDTA, 0.1% Triton X-100). After inactivation of the enzyme for 10 min at 95°C, a PCR was performed with Kapa HiFi enzyme (Roche) using primers that were homologous to the Illumina Read 1 and 2 DNA regions adjacent to the barcodes and included indexes and the standard Illumina P5 and P7 attachment sequences, facilitating easy subsequent indexed analysis. PCR conditions were as follows: after an initial denaturation at 95°C for 3 min, 5 cycles of 98°C 30 sec / 72°C 25 sec were followed by 20 cycles of 98°C 20 sec / 72°C 25 sec, a final elongation at 72°C for 3 min, and a return to 4°C. PCR products were pooled and subjected to QIAquick PCR product purification (Qiagen), followed by Illumina sequencing with standard primers. The presence of barcodes in specific plate- and well-pools pinpointed the specific plate/well location of a mutant in the collection.

### Selection of converted mutants for the final mutant collection

For the final barcoded collection, one mutant per gene converted from the non-barcoded SGD Kan^R^ lineage and one converted mutant per gene from the non-barcoded SGD Cam^R^ lineage were selected, whenever possible. Only mutants that were mapped to a singular plate/well location in the picked mutant plates without any detectable contamination by a mutant exhibiting a different barcode were considered. Furthermore, only mutants showing insertion sites in the correct orientation and within a 10-base window of the expected genome position were candidates. Preference was given to mutants whose insertion sites were closest to expectations (most often, an exact match), and those that had higher read numbers. Mutants that exhibited double resistance (Tet^R^ + Cam^R^ or Tet^R^ + Kan^R^) were excluded from consideration. Selected mutants were robotically picked from the 96-well picked plates, using a Biomek FX (Beckman), rearranged into their final assembly, regrown in LBTet^15^ and either stocked at -80°C in 20% glycerol or dried overnight at 30°C in 20% sucrose solution followed by storage at -80°C.

### Hyper-competency tests of select mutants in cell envelope genes

To investigate the competency of mutants that had been found to be hyper-convertible during the first round of conversions, 7 μl from overnight cultures of three of the following mutants were mixed into 21 ml of LB as follows: each of three mixes contained nalidixic acid-resistant STM14028s, one of three kanamycin-resistant mutant clones (Δ*ompH*, Δ*envZ* or Δ*bamB*) and one of three chloramphenicol-resistant mutant clones (Δ*ompC*, Δ*ompR* or ΔSTM14_4212-STM14_4225, a multi-gene deletion mutant encompassing *envZ* and *ompR*). Electrocompetent cells were then prepared after growth of these mixes in LB at 37°C to an OD of about 0.48, following standard protocols. An aliquot of the electrocompetent cell mixtures was used in a dilution series and plated on selective LB agar plates to test for similar growth and survival of the different mutants in each mix. Subsequently, the three mixes were directly electroporated with 100 ng of plasmid pKD46 (Amp^R^). Aliquots were plated immediately after electroporation and after a 50-min recovery period in SOC medium at 37°C onto selective LB agar plates supplemented with 100 μg/ml ampicillin, 80 μg/ml kanamycin, 20 μg/ml chloramphenicol, and/or 50 μg/ml nalidixic acid, as appropriate. Colonies were counted after overnight incubation.

### Motility test of the barcoded mutant collection to verify clone veracity

As an exemplar biological test, we performed a motility assay of our pooled barcoded mutants on 0.25% LB Tet^15^ agar plates. Briefly, 6–10 μl of a frozen stock of the pooled collection (about 10^7^ CFUs) were spotted directly onto the centers of agar plates, with subsequent growth in a 37°C incubator for 2 h, followed by an overnight incubation at room temperature. Alternatively, plates were incubated for about 6 h at 37°C. Growth proceeded until a visible ring had formed about 3 cm from the plate’s center, marking the expansion of standard motile bacteria from the inoculation site. Bacterial growth was collected from the center and the ring areas, using sterile cut pipet tips, resuspended in 3 ml LB Tet^15^, and grown overnight at 37°C with aeration. The experiment was performed four times on two different days.

Subsequently, 40 μl of the overnight growth was subjected to sample preparation for Illumina sequencing, as described above, using indexed Illumina primers. The frequency of each barcode was determined by analysis of Illumina sequencing readouts using custom Perl scripts (available upon request). Statistical relevance of changes in the representation of mutant clones was determined using DESeq2 [36].

## Results

### Workflow of the mutant resource conversion

**Fig 1:**
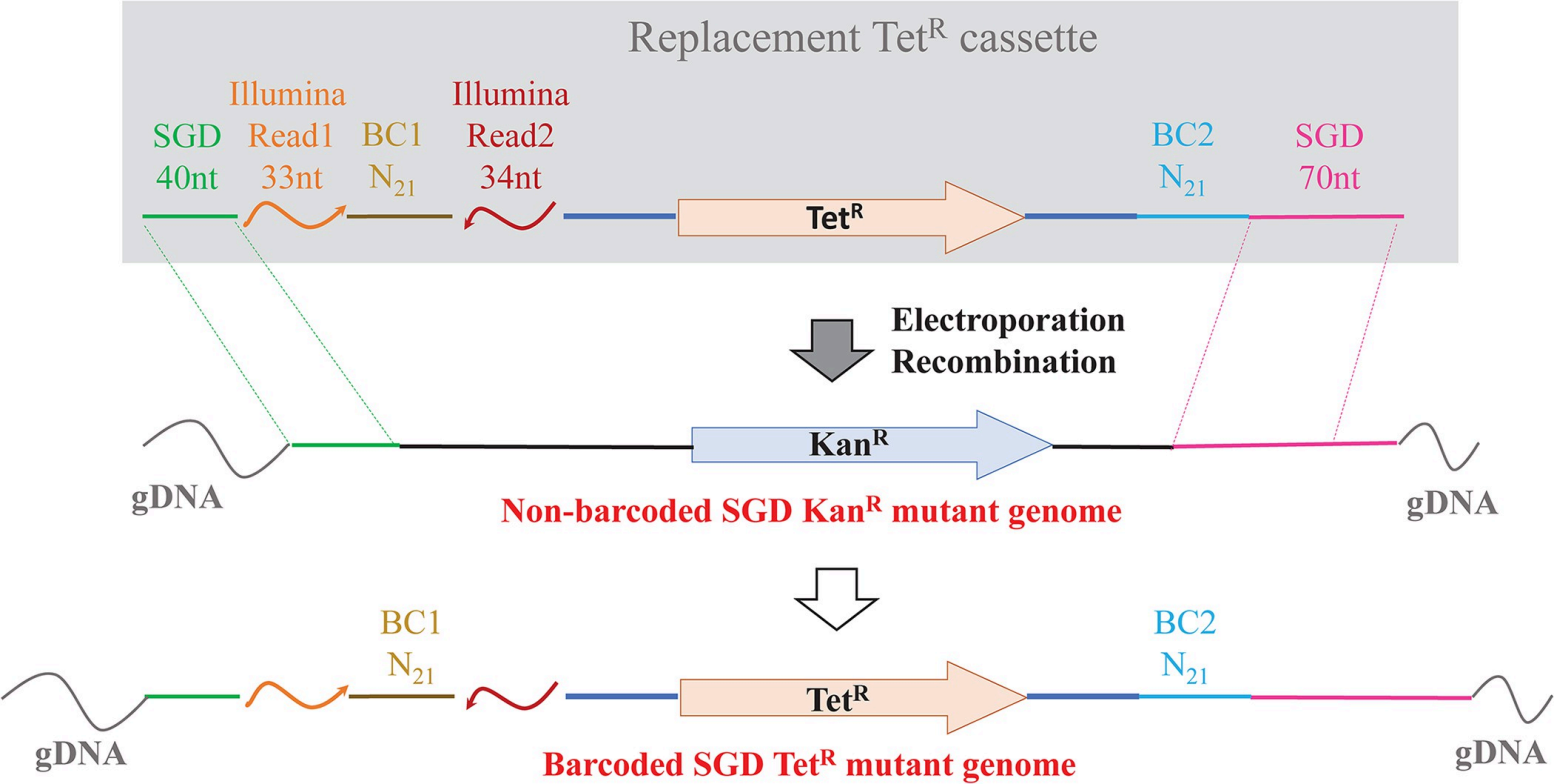
Lambda red recombination of barcodes into existing single-gene deletion mutants. A PCR product containing homology to both ends of the existing mutant cassette of non-barcoded mutants (green and pink lines) is recombined to produce tetracycline-resistant mutant clones with 21-base barcodes (BC1 and BC2), one of which is flanked by Illumina Read sequences. See **S1 Fig** for the complete sequence of the replacement cassette, and **Materials and Methods** for details.

The antibiotic resistance cassettes in an existing non-barcoded collection of Kan^R^ and Cam^R^ single gene deletion mutants in STM14028s were converted to a tetracycline resistance cassette (Tet^R^) using regions of homology at both ends of the cassettes. The introduced Tet^R^ cassette also encoded Illumina Read 1 and Read 2 sequences flanking a 21-mer barcode (BC1) and a second 21-base barcode (BC2), placed at the other end of the recombination product (**Fig 1**).

**Fig 2:**
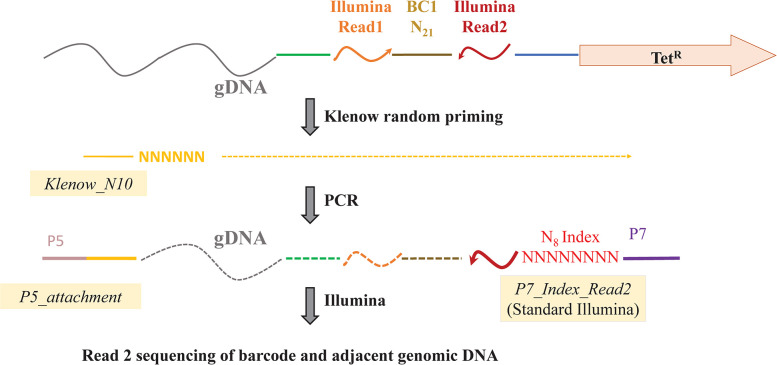
Workflow of the iterative conversion of the non-barcoded single-gene deletion (SGD) resource to barcoded Tet^R^ SGD collection. Conversion of the Kan^R^ resource is depicted. See text for details.

The mutant resource conversion was an iterative process that is illustrated in **Fig 2**. It included the following steps: First, each mutant in the existing non-barcoded SGD Kan^R^ and Cam^R^ collection was grown separately in 96-well plates and then each library was pooled separately. The two resulting pools were each made electrocompetent and electroporated with the pKD46 lambda red plasmid (Amp^R^). Second, a standard lambda red protocol was applied where the pools of transformants were grown at 30°C in 100 ug/ml ampicillin and then induced with arabinose to express the lambda red system [37]. Third, each pool of pKD46-containing cells was made electrocompetent and transformed with the replacement PCR product cassette encoding *tetR*. Each amplified Tet^R^ cassette molecule contained two of 4^21^ (4 trillion) possible different N_21_ barcodes, resulting in integration of unique and specific barcodes into each converted mutant genome.

**Fig 3:**
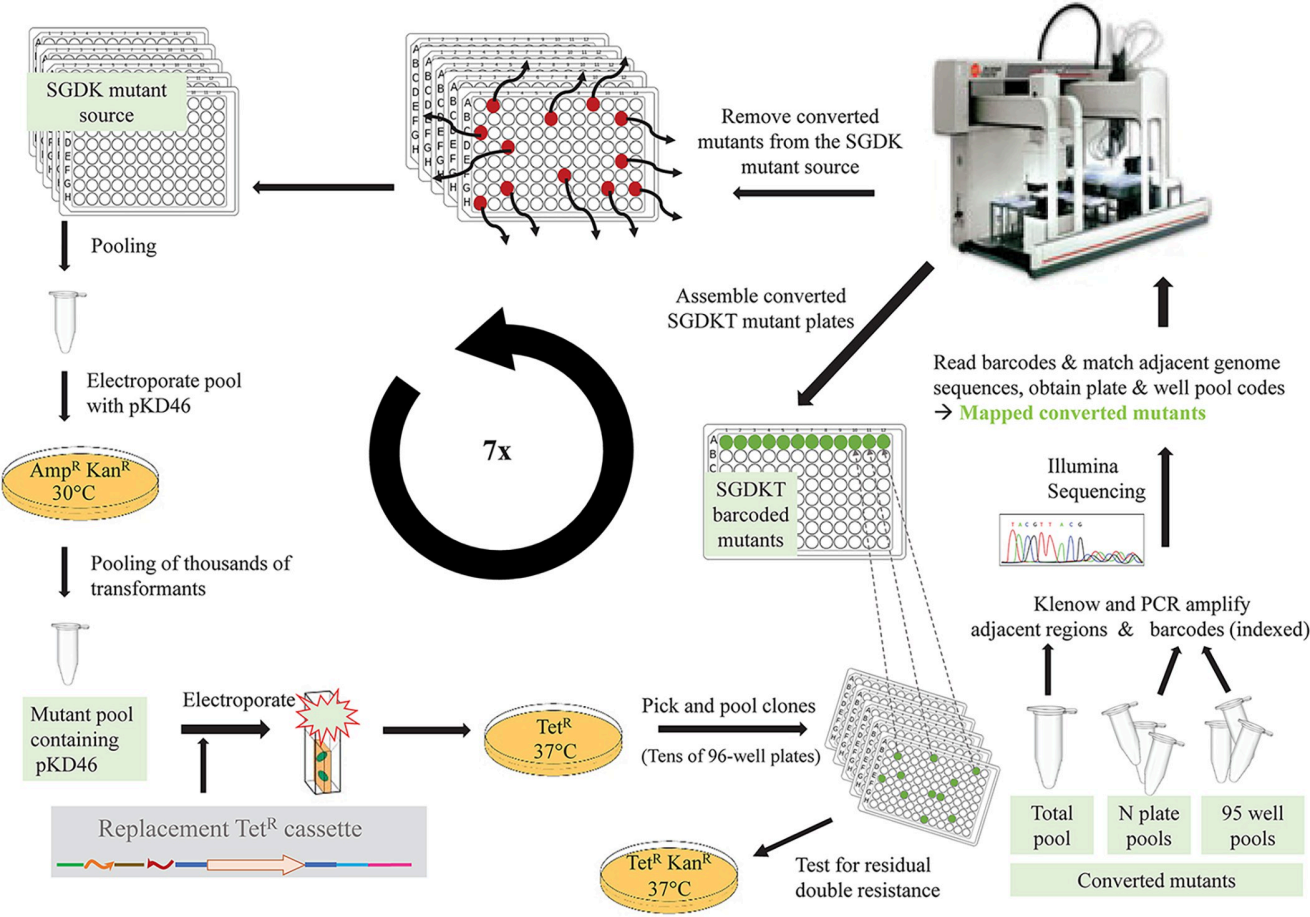
Determination of integration sites associated with specific barcodes in SGD libraries by Illumina sequencing after Klenow / PCR amplification. See **Materials and Methods** for details, including primer sequences.

Fourth, individual Tet^R^ colonies were picked into 96-well plates. For identification of successfully converted mutants, DNA was purified from a pool of all clones, denatured, and subjected to random primed synthesis using *E*. *coli* Klenow polymerase, followed by PCR using the specific portion of this primer and a standard Illumina Read 2 engraftment primer to amplify only the region of interest spanning the barcode and *Salmonella* DNA sequence adjacent the insert (**Fig 3**). Following Illumina sequencing of over a million reads and mapping to the genome, if at least 20 barcoded reads aligned in the correct orientation within a 5-base window to expected genome locations of mutant insertions, these reads were deemed to have been generated from that expected mutant.

The tetracycline-resistant mutants were replica-plated from agar plates into 96-well plates and tested for kanamycin or chloramphenicol resistance. Over 90% of converted Tet^R^ mutants exhibited neither Kan^R^ nor Cam^R^, indicating a successful swap of the resistance cassettes. Subsequently, the plate/well location of each picked clone was determined by pooling each 96-well plate of Tet^R^ mutants, separately (plate pools), and pooling the same well positions from all Tet^R^ mutant plates (well pools A01 through H11), using a Biomek FX liquid handling robot (Beckman). Aliquots of each of these pools were used as template for PCR using Illumina primers flanking the N_21_ barcode (see **Materials and Methods**). The barcodes, with known *Salmonella* locations determined in step 2, were mapped to plate/well locations. Mutants that were converted from the non-barcoded Kan^R^ mutant resource were kept separate from those that were converted from the non-barcoded Cam^R^ mutant resource.

Fifth, mutants that had been successfully converted were robotically removed from the plates containing the original non-barcoded collections. We then prepared competent cells from pools of these diminishing numbers of non-barcoded mutants. These competent cells were subjected to the same procedure (steps 1–5), a process that was repeated until over 90% of all non-barcoded SGD mutants had been converted. This goal was obtained after seven iterative conversion rounds.

In the final annealing step of producing the PCR product, strands with different barcodes may inadvertently anneal to each other, resulting in a “bubble” or “eye” within the double-stranded PCR product where each strand of the barcode portion is different. Consequently, upon replacement of the original non-barcoded cassette in a mutant, daughter mutants occasionally arose from the same conversion event that contained two different barcodes. This was detected when two barcodes of Tet^R^ bacteria residing in the same plate / well location mapped to the same genomic integration site. We avoided picking these bacteria for inclusion in our final collection and retained only one such case.

The number of different converted mutants found at each iteration is reported in **Table 2**. Interestingly, in iteration 01, efficiency (computed from the number of mutants in the pool, the number of TetR clones picked, and the number of converted mutants obtained) was low because there were a number of hyper-convertible mutants represented at high frequencies in the barcoded mutants (explained in more detail later). Efficiency was increased after these mutants had been removed from the source non-barcoded collections, and then decreased in later iterations perhaps because mutants that were difficult to convert, or grew more slowly, or had been incorrectly mapped in the source collections, accumulated in the later iterations.

**Table 2:** Number of clones picked and non-barcoded mutants successfully converted in each iteration of the conversion strategy.

Iteration	# of non-barcoded source clones	# of Tet^R^ colonies surveyed	# of different new Tet^R^ conversions obtained	Efficiency*
**SGDK conversions**				
Iteration01	4115	3135	774	0.35
Iteration02	3207	1330	788	0.72
Iteration03	2359	2090	794	0.57
Iteration04	1571	1900	552	0.50
Iteration05	1019	1900	302	0.35
Iteration06**	374	950	137	0.40
Iteration07	237	950	67	0.29
**SGDC conversions**				
Iteration01	4094	2090	496	0.30
Iteration02	3420	1045	682	0.76
Iteration03	2691	1140	489	0.53
Iteration04	2202	2090	693	0.51
Iteration05	1509	1900	365	0.34
Iteration06**	635	1425	226	0.40
Iteration07	409	1140	62	0.16

* Number of different new Tet^R^ conversions obtained with a binomial adjustment for the number of Kan^R^ or Cam^R^ colonies surveyed, and Tet^R^ clones picked.

** For iteration 06, only the remaining unconverted mutants with known plate/well locations were used, causing a jump in conversion efficiency.

### Characteristics of the final collection of barcoded single-gene deletion mutants

The final barcoded Tet^R^ STM14028s collection is organized in sixty-six 96-well plates. A total of 3267 barcoded mutants that had been converted from Kan^R^ non-barcoded clones are organized in 3246 wells of plates SGDKT-01 through SGDKT-35 (NRS59536 –NRS59570 at beiresources.org), while 2940 barcoded mutants that had been converted from Cam^R^ non-barcoded clones are organized in 2913 wells of plates SGDCT-01 through SGDCT-31 (NRS59504 –NRS59534).

Based on the GenBank annotation of the STM14028s genome (GCA_000022165.1), mutants in 63.8% of all annotated CDSs (3490 / 5474) are present in the barcoded collection, with 67% of those (a total of 2355 CDSs) represented by at least two (and up to five) independently barcoded mutants, of which 2347 included at least one each from the kanamycin-resistant lineage and the chloramphenicol-resistant lineage of non-barcoded SGD clones. **S2 Table** shows the characteristics of barcoded mutants for each annotated feature of the genome. A total of 3184 genome features in the non-barcoded Kan^R^ library and 2877 features in non-barcoded Cam^R^ library were converted to barcoded Tet^R^. A few mutants present in the non-barcoded libraries and detected during the iterative conversion process did not make it into the final barcoded set due to uncertain mapping of their plate/well location in the plates from which the final sets were picked, contamination in the same well, or double resistance stemming from a failed swapping event. This occurred for about 6.7% of originally identified barcoded SGDKT mutants, and 4.5% of barcoded SGDCT mutants. Conversely, the STM14_4922 (*cytR*) mutant that existed but at unknown plate/well locations in the non-barcoded collections, was converted and mapped in the barcoded collection.

A final tally of the conversion of non-barcoded mutants showed that 3535 of 3878 mutants (91.2%) had been successfully converted with our iterative strategy (**Table 3**). All members of the unbarcoded Kan^R^ and Cam^R^ mutant collections [27, 28] and their conversion to barcoded Tet^R^ are shown in **S3 Table**. All barcodes present in the final barcoded collection, their corresponding genome insertion location and plate/well localization are presented in **S4 Table**.

**Table 3:** Yield of barcoded mutants.

	Kan^R^	Cam^R^	Kan^R^ OR Cam^R^	Kan^R^ AND Cam^R^
Number of non-barcoded mutants (source)	3612	3463	3876	3199
Number of converted Tet^R^ barcoded mutants	3064	2770	3535	2299
Percent conversion	84.8	80.0	91.2	71.9

### Identification of genes with a possible role in DNA uptake

During the first iteration of our conversion process, some existing non-barcoded mutants had a strongly elevated frequency of conversion to Tet^R^. **Table 4** lists mutants that occurred at a rate of over 1% of all conversions. For example, ΔSTM14_0267 (*ompH*) contributed an astonishing 31% of all barcodes detected in the first conversion iteration of the non-barcoded Kan^R^ resource. Notably, five of the eight genes falling into this category (*ompH*, *bamB*, *ompC*, *envZ* and *ompR*) are intimately involved in outer membrane protein assembly or function. After removal of these mutants, the second conversion iteration was highly efficient.

**Table 4:** Mutants with a conversion frequency of greater than 1% in the first iteration.

			Kan^R^ conversion, 1^st^ iteration		Cam^R^ conversion, 1^st^ iteration	
Gene number	Gene name	Genome location of insert	Number of different barcodes detected (≥ 5 counts)	% Contribution to total number of different barcodes (≥ 5 counts)	Number of different barcodes detected (≥ 5 counts)	% Contribution to total number of different barcodes (≥ 5 counts)
STM14_0267	*hlpA (ompH)*	265650	929	**31.07**	no SGDC mutant	
STM14_3091	*yfgL (bamB)*	2705749	195	**6.52**	no SGDC mutant	
STM14_2797	*ompC*	2418103	143	**4.78**	38	**5.86**
STM14_4216	*envZ*	3673478	135	**4.52**	no SGDC mutant	
STM14_4217	*ompR*	3674194	115	**3.85**	6	0.92
STM14_1387	*ycfQ*	1255320	41	**1.37**	18	**2.77**
STM14_3570	*gudT*	3134376	1	0.03	12	**1.85**
STM14_4033	*sspA*	3525058	8	0.27	7	**1.08**

One possibility was that these mutants represented at a higher proportion in each non-barcoded library pool prior to the electroporation with pKD46. However, this was unlikely because the pools were constructed from separate growths of each mutant, followed by mixing and limited growth to early log phase to produce the electrocompetent cells. The ability of these five different gene deletions to be transformed with plasmid pKD46 by electroporation was tested in a mixture with wild type STM14028s. Three-component cell mixtures each contained nalidixic acid-resistant STM14028s as a control, one of three kanamycin-resistant mutant clones (Δ*ompH*, Δ*envZ* or Δ*bamB*) and one of three chloramphenicol-resistant mutant clones (Δ*ompC*, Δ*ompR* or ΔSTM14_4212-STM14_4225, a multi-gene deletion mutant encompassing *envZ* and *ompR*). Plating following each step of the electroporation procedure (after preparation of competent cells, directly after electroporation, and after 50 minutes of recovery in SOC media at 37°C) confirmed that after recovery in SOC and after taking into account their representation in the competent cells, the mutant clones yielded more transformants than the wild type cells. Increases in transformation efficiency ranged from a 1.5-fold relative to wild type (for Δ*ompC*), 5-fold (Δ*ompR*, Δ*envZ*, Δ STM14_4212-STM14_4225), 15-fold (Δ*ompH*), and 50-fold (Δ*bamB*). Increased efficiency of transformation was already visible when plating immediately after electroporation prior to recovery in SOC medium for the Δ*ompR* and Δ*envZ* strains (5-fold), the Δ*ompH* strain (7.5-fold) and the Δ*bamB* strain (10-fold). These experiments suggested an increased ability of these five mutants to accept supercoiled plasmid DNA during electroporation.

### Use of the barcoded resource in a biological experiment–motility assay

**Fig 4:**
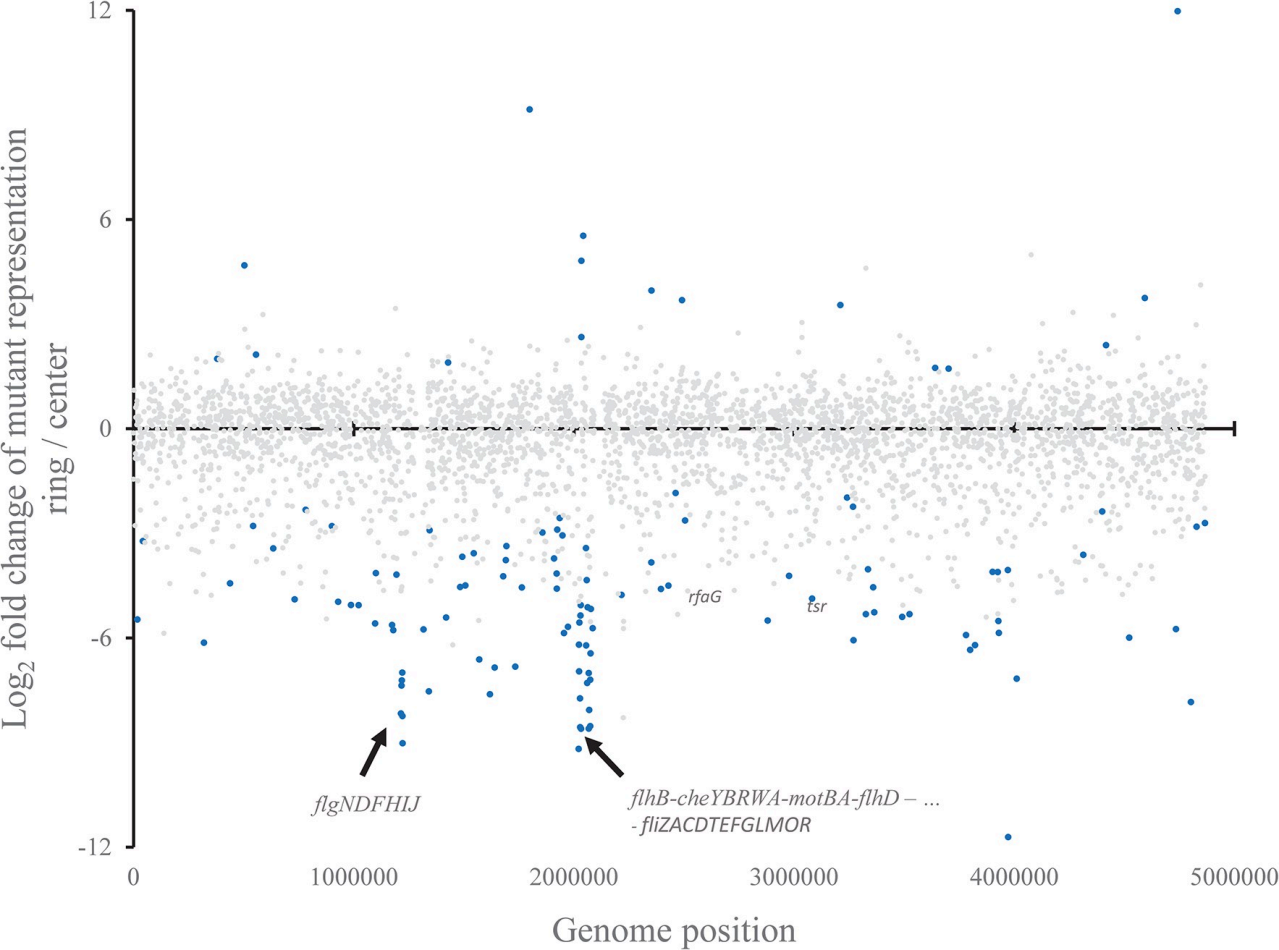
Differences in mutant representation in the ring vs the center of motility agar plates after inoculation with a pool of 6,207 barcoded single-gene deletion mutants. See **Materials & Methods** for details. Each dot depicts a gene, usually represented by two mutants which were combined in the statistical analysis. Underlying data in single-mutant resolution are shown in ***S5* Table**. Blue = p_adj_ < 0.05; grey = p_adj_ > 0.05. Some notable statistically significant genes are annotated. Data are from four separate experiments.

We performed a motility test to verify the ability of the barcoded SGD collection, when used as a pool, to reveal candidate mutants that play a role in a specific process. A total of 6–10 μl of a frozen stock of a pool of all barcoded Tet^R^ mutants was inoculated on the surface of 0.25% LB Tet agar “swim” plates. Plates were incubated at 37°C or at room temperature until a circle of motile bacteria had developed around the inoculation site and a clearly visible front line of motile bacteria had migrated approximately 5 cm from the center. The experiment was performed four times on two different days. Agar was collected from the inoculation site (where non-motile and residual motile bacteria were located) and from the front line (where mostly motile bacteria were located), pelleted and prepared for sequencing in a one-tube processing followed by PCR (see **Materials and Methods**). Sequencing data were processed and statistical analyses of a comparison of the mutants present in the center with those present in the ring revealed that flagellar and chemotaxis mutants were heavily underrepresented in the ring fraction. **Fig 4** depicts the ratios of representation in the ring over the center obtained for each gene represented by the barcoded collection of mutants, sorted by gene location on the STM14028s chromosome. As expected, the top 10 genes that were underrepresented in the ring fraction compared to the center were annotated with motility features. Overall, 23 of the 58 most underrepresented genes (adjusted p < 0.01) are annotated with flagellar or chemotaxis functions. An analysis in single mutant resolution identified 205 mutants at p_adj_ < 0.05, representing 187 different chromosomal elements, the vast majority of which (184 mutants) were underrepresented in the ring and therefore restricted in their motility while 21 were overrepresented in the ring fraction. 17 genes whose mutants were under selection were represented by more than one mutant in this set, and in each case, the change of the representation was in the same direction (up or down), suggesting that the behavior of independent Tet^R^ mutants in the same gene was consistent. **S5 Table** depicts the ratios and adjusted p values of all 6206 barcodes denoting mutants in the Tet^R^ barcoded SGD collection in the two fractions (ring versus center) harvested from the motility agar plates.

### Collection uses, availability, and alerts

The new barcoded mutant resource can be used to generate custom pools of desired mutants for low-complexity experiments. In addition to subsets being available from us via collaboration, the entire barcoded collection has been deposited for distribution at BEI Resources (www.beiresources.org), with plate identifiers NRS59504 –NRS59534 (SGDCT-01 through SGDCT-31) and NRS59536 –NRS59570 (SGDKT-01 through SGDKT-35).

Barcoded deletion mutants may have second site mutations that alter their phenotypes. These effects can be substantial, as observed for the *sirA/barA* two-component regulatory system, where mutations markedly increase *Salmonella*’s persistence in humans and mice [38]. We retained two independent deletion mutants for the majority of genes so that unwanted secondary mutations may be detected if the two mutants behave differently in investigated conditions. After mutants are identified as potentially relevant in an experiment, we strongly recommend transfer into a clean background when follow-up studies are performed.

We have identified 61 cases of cross-contamination in the 6159 wells of our collection that likely contain more than one barcoded mutant (**S2 Table**). There may be additional contamination at lower levels. In general, mutants with a phenotype should be confirmed by PCR and by transduction.

## Discussion

Genome-wide defined single-gene deletion mutant collections are useful for efficiently finding or confirming mutant phenotypes and, if multiple antibiotic resistance markers are available, for the assembly of double mutants. However, the costs of such a resource are rarely funded by the usual grant opportunities and therefore construction of genome-wide bacterial SGD collections has largely been restricted to important model organisms such as *E*. *coli* [23, 24], *Bacillus subtilis* [25], or *Salmonella enterica* sv Typhimurium [27, 28]. When available, these resources have facilitated a wealth of biological insights evidenced by hundreds of publications.

We present a barcoded single-gene deletion resource for *Salmonella enterica* sv Typhimurium 14028s which we have derived from an existing non-barcoded mutant collection. The entire non-barcoded collection was used as a recipient of a barcoded resistance cassette designed to integrate as a swap-out product to replace the existing cassettes. In an iterative process, successfully converted and confirmed mutants were removed from the non-barcoded collection prior to the next round of conversion (**Fig 2**). This way, we barcoded single-gene deletion mutants representing over 90% of previously deleted genes in seven rounds of conversion for the prior resources (Kan^R^ and Cam^R^). This strategy was in contrast to the over 7,000 PCRs and electroporations and several person-years that were used to generate the original non-barcoded resource from a wild type bacterium [27, 28]. The iterative conversion strategy was quicker and cheaper, even though it required identification of converted mutants and their plate/well location in picked 96-well plates after each round.

Collections of transposon or single-gene deletion mutants can be screened as pools, relying on protocols that amplify the adjacent host DNA. A barcoded version of such a collection of mutants, flanked by Illumina Read sequences, is an improvement that dramatically simplifies the protocol of preparing pooled DNAs for sequencing (**S1 Table,** [10, 11]). Indeed, here we present a simplified protocol of single-tube processing of crude bacterial extracts followed by a single-step standard PCR and Illumina sequencing, with much reduced effort and cost during downstream analysis. In addition, minimal ordered sets of barcoded mutants that maximize coverage of genes of interest can be screened under conditions that preclude the use of highly complex mixtures [39]. Restricted access of a pathogen occurs often in biological models such as animal studies where passage of bacteria through various bottlenecks can be limited to a few hundred founder cells that subsequently expand once they have reached a more favorable niche.

During library conversion we observed that certain mutants were more highly receptive to transformation than most mutants (**Table 4**). These included mutants in five genes involved in outer membrane protein assembly, namely *ompH*, *bamB*, *ompC*, *envZ* and *ompR*. The results for these five genes were confirmed in separate competition experiments with the parental strain. The *ompH* mutant had the highest confirmed competence for plasmid transformation. The gene encodes a 16-kDa outer membrane protein [40], and functions as a periplasmic chaperone that is involved in the periplasmic transit and membrane insertion of several proteins in the membrane [41, 42]. Therefore, membrane permeability may have been increased in this mutant, possibly allowing entry of DNA into the cells at an increased rate. Similar effects may have caused the observed hyper-convertibility for the mutants of the other four genes involved in outer membrane composition. BamB has been found to affect outer membrane permeability of the bacterium to antibiotics [43, 44], while the two-component system EnvZ/OmpR controls the expression of several porins including OmpC, OmpF [45], and others [46]. It may be interesting to test whether deletion of any of these genes, in combination with other known gene defects, would also dramatically increase competency of commercially available electrocompetent *E*. *coli* cells.

The utility of the resulting barcoded collection was verified in a model experiment that compared motile bacteria on a swim plate with a population sampled from the area near the inoculation site. As expected, the analysis revealed that mutants in genes with a known role in bacterial motility were overrepresented in the inoculated center of the motility plate, compared with the location of motile bacteria. Nearly all mutants in flagellar and chemotaxis genes were present at higher representation in the population sampled from the center (**Fig 4, S5 Table**), confirming the veracity of the barcoded collection.

In conclusion, the introduction of unique barcodes, one of which is flanked by Illumina Read sequences, into our single-gene deletion resource allows researchers to perform screening experiments at much-reduced cost and effort, and enhances their ability to create reduced-complexity subset pools of mutants.

## Supporting information

S1 Table Random and ordered barcoded transposon libraries produced in our lab. (XLSX)

S2 Table All annotated features of the STM14028s genome and corresponding barcoded clones. (XLSX)

S3 Table Barcode conversion data for the non-barcoded single-gene deletion mutants reported in [28]. (XLSX)

S4 Table Annotation and plate/well location of all barcodes identified in the barcoded STM14028s SGD collection. (XLSX)

S5 Table Differences in mutant representation on motility agar in the motile ring and inoculation center. (XLSX)

S1 Fig Map, sequence, and features of the barcoded Tet^R^ resistance cassette. (TIF)

## References

[1] Broz P, Ohlson MB, Monack DM. Innate immune response to *Salmonella typhimurium*, a model enteric pathogen. Gut Microbes. 2012;3(2):62–70. 22198618 10.4161/gmic.19141 PMC3370950

[2] Ohl ME, Miller SI. *Salmonella*: a model for bacterial pathogenesis. Annu Rev Med. 2001;52:259–74. 11160778 10.1146/annurev.med.52.1.259

[3] Tsolis RM, Kingsley RA, Townsend SM, Ficht TA, Adams LG, Bäumler AJ. Of mice, calves, and men. Comparison of the mouse typhoid model with other *Salmonella* infections. Adv Exp Med Biol. 1999;473:261–74. 10659367

[4] Galan JE. *Salmonella* Typhimurium and inflammation: a pathogen-centric affair. Nat Rev Microbiol. 2021;19(11):716–25. 34012042 10.1038/s41579-021-00561-4 PMC9350856

[5] Hajra D, Nair AV, Chakravortty D. Decoding the invasive nature of a tropical pathogen of concern: The invasive non-Typhoidal *Salmonella* strains causing host-restricted extraintestinal infections worldwide. Microbiol Res. 2023;277:127488. 37716125 10.1016/j.micres.2023.127488

[6] Botstein D, Maurer R. Genetic approaches to the analysis of microbial development. Annual review of genetics. 1982;16:61–83. doi: 10.1146/annurev.ge.16.120182.000425 6297378

[7] Cain AK, Barquist L, Goodman AL, Paulsen IT, Parkhill J, van Opijnen T. A decade of advances in transposon-insertion sequencing. Nat Rev Genet. 2020;21(9):526–40. doi: 10.1038/s41576-020-0244-x 32533119 PMC7291929

[8] Hensel M, Shea JE, Gleeson C, Jones MD, Dalton E, Holden DW. Simultaneous identification of bacterial virulence genes by negative selection. Science. 1995;269(5222):400–3. doi: 10.1126/science.7618105 7618105

[9] Saenz HL, Dehio C. Signature-tagged mutagenesis: technical advances in a negative selection method for virulence gene identification. Curr Opin Microbiol. 2005;8(5):612–9. doi: 10.1016/j.mib.2005.08.013 16126452

[10] de Moraes MH, Desai P, Porwollik S, Canals R, Perez DR, Chu W, et al. *Salmonella* persistence in tomatoes requires a distinct set of metabolic functions identified by transposon insertion sequencing. Appl Environ Microbiol. 2017;83(5). 10.1128/AEM.03028-16 PMC5311394 28039131

[11] Jayeola V, McClelland M, Porwollik S, Chu W, Farber J, Kathariou S. Identification of novel genes mediating survival of *Salmonella* on low-moisture foods via transposon sequencing analysis. Front Microbiol. 2020;11:726. 32499760 10.3389/fmicb.2020.00726 PMC7242855

[12] Fey PD, Endres JL, Yajjala VK, Widhelm TJ, Boissy RJ, Bose JL, et al. A genetic resource for rapid and comprehensive phenotype screening of nonessential *Staphylococcus aureus* genes. mBio. 2013;4(1):e00537–12. 23404398 10.1128/mBio.00537-12 PMC3573662

[13] Bae T, Banger AK, Wallace A, Glass EM, Aslund F, Schneewind O, et al. *Staphylococcus aureus* virulence genes identified by bursa aurealis mutagenesis and nematode killing. Proc Natl Acad Sci U S A. 2004;101(33):12312–7. 15304642 10.1073/pnas.0404728101 PMC514475

[14] Mike LA, Stark AJ, Forsyth VS, Vornhagen J, Smith SN, Bachman MA, et al. A systematic analysis of hypermucoviscosity and capsule reveals distinct and overlapping genes that impact *Klebsiella pneumoniae* fitness. PLoS Pathog. 2021;17(3):e1009376. 33720976 10.1371/journal.ppat.1009376 PMC7993769

[15] Shea AE, Marzoa J, Himpsl SD, Smith SN, Zhao L, Tran L, et al. *Escherichia coli* CFT073 Fitness Factors during Urinary Tract Infection: Identification Using an Ordered Transposon Library. Appl Environ Microbiol. 2020;86(13). 10.1128/AEM.00691-20 PMC7301846 32358013

[16] Dale JL, Beckman KB, Willett JLE, Nilson JL, Palani NP, Baller JA, et al. Comprehensive functional analysis of the *Enterococcus faecalis* core genome using an ordered, sequence-defined collection of insertional mutations in strain OG1RF. mSystems. 2018;3(5). 10.1128/mSystems.00062-18 PMC6134198 30225373

[17] Cameron DE, Urbach JM, Mekalanos JJ. A defined transposon mutant library and its use in identifying motility genes in *Vibrio cholerae*. Proc Natl Acad Sci U S A. 2008;105(25):8736–41. 18574146 10.1073/pnas.0803281105 PMC2438431

[18] Liberati NT, Urbach JM, Miyata S, Lee DG, Drenkard E, Wu G, et al. An ordered, nonredundant library of *Pseudomonas aeruginosa* strain PA14 transposon insertion mutants. Proc Natl Acad Sci U S A. 2006;103(8):2833–8. 16477005 10.1073/pnas.0511100103 PMC1413827

[19] Held K, Ramage E, Jacobs M, Gallagher L, Manoil C. Sequence-verified two-allele transposon mutant library for *Pseudomonas aeruginosa* PAO1. J Bacteriol. 2012;194(23):6387–9. 22984262 10.1128/JB.01479-12 PMC3497512

[20] Vandewalle K, Festjens N, Plets E, Vuylsteke M, Saeys Y, Callewaert N. Characterization of genome-wide ordered sequence-tagged *Mycobacterium* mutant libraries by Cartesian Pooling-Coordinate Sequencing. Nat Commun. 2015;6:7106. 25960123 10.1038/ncomms8106 PMC4432585

[21] Borgers K, Vandewalle K, Van Hecke A, Michielsen G, Plets E, van Schie L, et al. Development of a Counterselectable Transposon To Create Markerless Knockouts from an 18,432-Clone Ordered *Mycobacterium bovis* Bacillus Calmette-Guérin Mutant Resource. mSystems. 2020;5(4). 10.1128/mSystems.00180-20 PMC7426150 32788404

[22] Lin T, Gao L, Zhang C, Odeh E, Jacobs MB, Coutte L, et al. Analysis of an ordered, comprehensive STM mutant library in infectious *Borrelia burgdorferi*: insights into the genes required for mouse infectivity. PLoS One. 2012;7(10):e47532. 23133514 10.1371/journal.pone.0047532 PMC3485029

[23] Yamamoto N, Nakahigashi K, Nakamichi T, Yoshino M, Takai Y, Touda Y, et al. Update on the Keio collection of *Escherichia coli* single-gene deletion mutants. Mol Syst Biol. 2009;5:335. 20029369 10.1038/msb.2009.92 PMC2824493

[24] Baba T, Ara T, Hasegawa M, Takai Y, Okumura Y, Baba M, et al. Construction of *Escherichia coli* K-12 in-frame, single-gene knockout mutants: the Keio collection. Mol Syst Biol. 2006;2:2006 0008. 10.1038/msb4100050 PMC1681482 16738554

[25] Koo BM, Kritikos G, Farelli JD, Todor H, Tong K, Kimsey H, et al. Construction and Analysis of Two Genome-Scale Deletion Libraries for *Bacillus subtilis*. Cell Syst. 2017;4(3):291–305 e7. 28189581 10.1016/j.cels.2016.12.013 PMC5400513

[26] Yang DC, Blair KM, Taylor JA, Petersen TW, Sessler T, Tull CM, et al. A Genome-Wide *Helicobacter pylori* Morphology Screen Uncovers a Membrane-Spanning Helical Cell Shape Complex. J Bacteriol. 2019;201(14). 10.1128/JB.00724-18 PMC6597387 31036730

[27] Santiviago CA, Reynolds MM, Porwollik S, Choi SH, Long F, Andrews-Polymenis HL, et al. Analysis of pools of targeted *Salmonella* deletion mutants identifies novel genes affecting fitness during competitive infection in mice. PLoS Pathog. 2009;5(7):e1000477. 19578432 10.1371/journal.ppat.1000477 PMC2698986

[28] Porwollik S, Santiviago CA, Cheng P, Long F, Desai P, Fredlund J, et al. Defined single-gene and multi-gene deletion mutant collections in *Salmonella enterica* sv Typhimurium. PLoS One. 2014;9(7):e99820. 25007190 10.1371/journal.pone.0099820 PMC4089911

[29] Silva-Valenzuela CA, Molina-Quiroz RC, Desai P, Valenzuela C, Porwollik S, Zhao M, et al. Analysis of two complementary single-gene deletion mutant libraries of *Salmonella* Typhimurium in intraperitoneal infection of BALB/c mice. Front Microbiol. 2015;6:1455. 26779130 10.3389/fmicb.2015.01455 PMC4700939

[30] Yang HJ, Bogomolnaya LM, Elfenbein JR, Endicott-Yazdani T, Reynolds MM, Porwollik S, et al. Novel two-step hierarchical screening of mutant pools reveals mutants under selection in chicks. Infect Immun. 2016;84(4):1226–38. doi: 10.1128/IAI.01525-15 26857572 PMC4807481

[31] Wrande M, Andrews-Polymenis H, Twedt DJ, Steele-Mortimer O, Porwollik S, McClelland M, et al. Genetic determinants of *Salmonella enterica* serovar Typhimurium proliferation in the cytosol of epithelial cells. Infect Immun. 2016;84(12):3517–26. 27698022 10.1128/IAI.00734-16 PMC5116728

[32] Kullas AL, McClelland M, Yang HJ, Tam JW, Torres A, Porwollik S, et al. L-asparaginase II produced by *Salmonella typhimurium* inhibits T cell responses and mediates virulence. Cell Host Microbe. 2012;12(6):791–8. 23245323 10.1016/j.chom.2012.10.018 PMC4361029

[33] Ellis MJ, Tsai CN, Johnson JW, French S, Elhenawy W, Porwollik S, et al. A macrophage-based screen identifies antibacterial compounds selective for intracellular *Salmonella* Typhimurium. Nat Commun. 2019;10(1):197. 30643129 10.1038/s41467-018-08190-x PMC6331611

[34] Ronneau S, Michaux C, Helaine S. Decline in nitrosative stress drives antibiotic persister regrowth during infection. Cell Host Microbe. 2023;31(6):993–1006 e6. doi: 10.1016/j.chom.2023.05.002 37236190

[35] Griewisch KF, Pierce JG, Elfenbein JR. Genetic Determinants of Salmonella Resistance to the Biofilm-Inhibitory Effects of a Synthetic 4-Oxazolidinone Analog. Appl Environ Microbiol. 2020;86(20). 10.1128/AEM.01120-20 PMC7531968 32769186

[36] Love MI, Huber W, Anders S. Moderated estimation of fold change and dispersion for RNA-seq data with DESeq2. Genome Biol. 2014;15(12):550. doi: 10.1186/s13059-014-0550-8 25516281 PMC4302049

[37] Datsenko KA, Wanner BL. One-step inactivation of chromosomal genes in *Escherichia coli* K-12 using PCR products. Proc Natl Acad Sci U S A. 2000;97(12):6640–5. 10829079 10.1073/pnas.120163297 PMC18686

[38] Grote A, Piscon B, Manson AL, Adani B, Cohen H, Livny J, et al. Persistent *Salmonella* infections in humans are associated with mutations in the BarA/SirA regulatory pathway. Cell Host Microbe. 2024;32(1):79–92 e7. 38211565 10.1016/j.chom.2023.12.001 PMC11410052

[39] Margolis A, Liu L, Porwollik S, Till JKA, Chu W, McClelland M, et al. Arginine metabolism powers *Salmonella* resistance to oxidative stress. Infect Immun. 2023;91(6):e0012023. 37191509 10.1128/iai.00120-23 PMC10269097

[40] Koski P, Hirvas L, Vaara M. Complete sequence of the ompH gene encoding the 16-kDa cationic outer membrane protein of *Salmonella typhimurium*. Gene. 1990;88(1):117–20. 2187745 10.1016/0378-1119(90)90068-3

[41] Harms N, Koningstein G, Dontje W, Muller M, Oudega B, Luirink J, et al. The early interaction of the outer membrane protein phoe with the periplasmic chaperone Skp occurs at the cytoplasmic membrane. J Biol Chem. 2001;276(22):18804–11. doi: 10.1074/jbc.M011194200 11278858

[42] Bulieris PV, Behrens S, Holst O, Kleinschmidt JH. Folding and insertion of the outer membrane protein OmpA is assisted by the chaperone Skp and by lipopolysaccharide. J Biol Chem. 2003;278(11):9092–9. doi: 10.1074/jbc.M211177200 12509434

[43] Ruiz N, Falcone B, Kahne D, Silhavy TJ. Chemical conditionality: a genetic strategy to probe organelle assembly. Cell. 2005;121(2):307–17. doi: 10.1016/j.cell.2005.02.014 15851036

[44] Namdari F, Hurtado-Escobar GA, Abed N, Trotereau J, Fardini Y, Giraud E, et al. Deciphering the roles of BamB and its interaction with BamA in outer membrane biogenesis, T3SS expression and virulence in *Salmonella*. PLoS One. 2012;7(11):e46050. 23144780 10.1371/journal.pone.0046050 PMC3489874

[45] Forst S, Delgado J, Inouye M. Phosphorylation of OmpR by the osmosensor EnvZ modulates expression of the ompF and ompC genes in *Escherichia coli*. Proc Natl Acad Sci U S A. 1989;86(16):6052–6. 2668953 10.1073/pnas.86.16.6052 PMC297773

[46] Ko D, Choi SH. Mechanistic understanding of antibiotic resistance mediated by EnvZ/OmpR two-component system in *Salmonella enterica* serovar Enteritidis. J Antimicrob Chemother. 2022;77(9):2419–28. 35781339 10.1093/jac/dkac223

