# Supplementary figures and images for: A genome-wide collection of barcoded single-gene deletion mutants in Salmonella enterica serovar Typhimurium
Source: PLoS One. 2024 Mar 7;19(3):e0298419. doi: 10.1371/journal.pone.0298419 (PMC10919679; doi:10.1371/journal.pone.0298419)

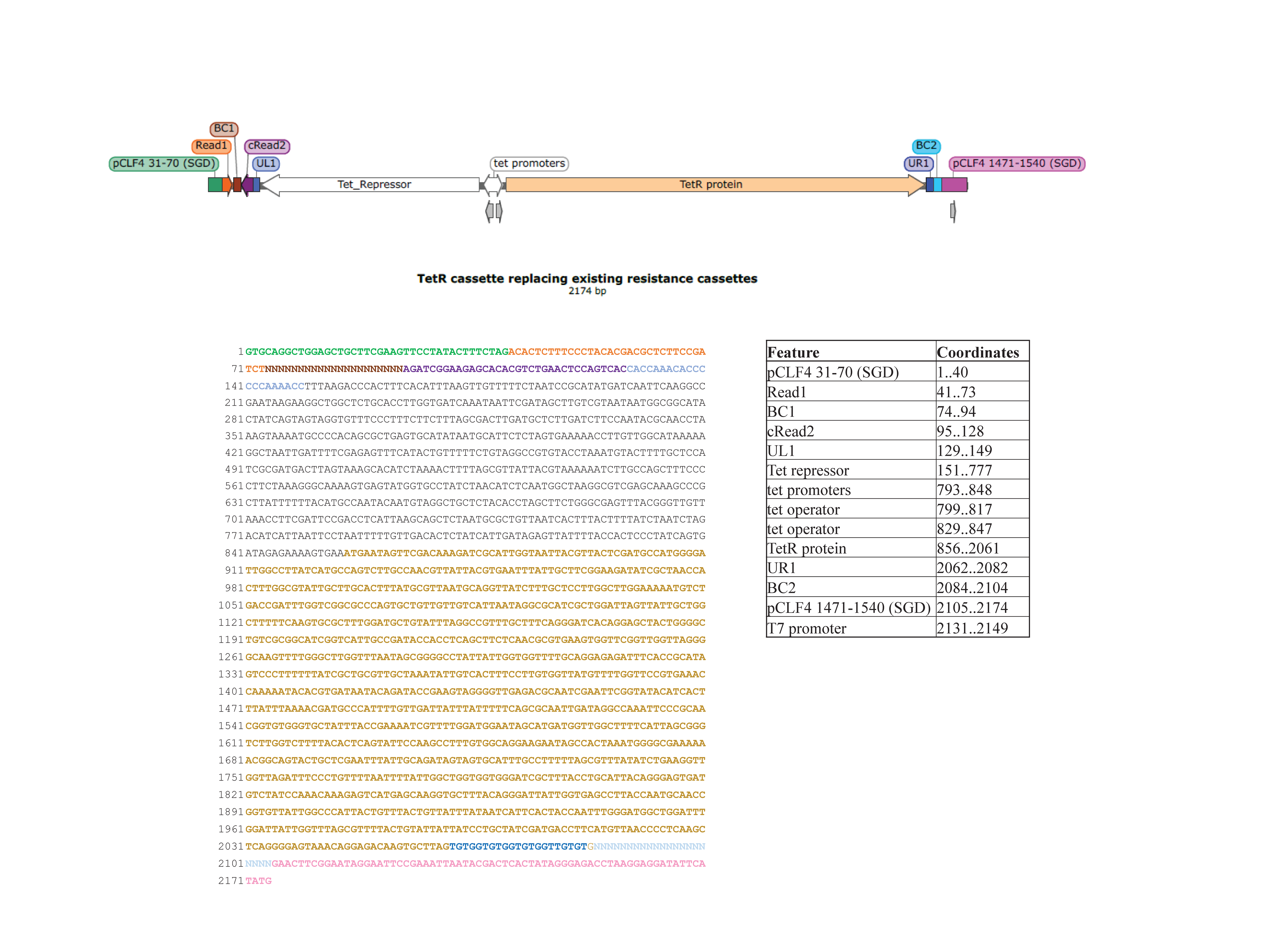

Supplement: S1 Fig — (TIF) [file pone.0298419.s006.tif]
